# Slow-Frequency Pulsed Transcranial Electrical Stimulation for Modulation of Cortical Plasticity Based on Reciprocity Targeting with Precision Electrical Head Modeling

**DOI:** 10.3389/fnhum.2016.00377

**Published:** 2016-08-02

**Authors:** Phan Luu, Easwara Moorthy Essaki Arumugam, Erik Anderson, Amanda Gunn, Dennis Rech, Sergei Turovets, Don M. Tucker

**Affiliations:** ^1^Electrical Geodesics, Inc., EugeneOR, USA; ^2^Department of Psychology, University of Oregon, EugeneOR, USA; ^3^NeuroInformatics Center, University of Oregon, EugeneOR, USA

**Keywords:** cortical plasticity, head tissue conductivity, transcranial electrical stimulation, transcranial direct current stimulation, transcranial alternating current stimulation, transcranial pulsed current stimulation

## Abstract

In pain management as well as other clinical applications of neuromodulation, it is important to consider the timing parameters influencing activity-dependent plasticity, including pulsed versus sustained currents, as well as the spatial action of electrical currents as they polarize the complex convolutions of the cortical mantle. These factors are of course related; studying temporal factors is not possible when the spatial resolution of current delivery to the cortex is so uncertain to make it unclear whether excitability is increased or decreased with anodal vs. cathodal current flow. In the present study we attempted to improve the targeting of specific cortical locations by applying current through flexible source-sink configurations of 256 electrodes in a geodesic array. We constructed a precision electric head model for 12 healthy individuals. Extraction of the individual’s cortical surface allowed computation of the component of the induced current that is normal to the target cortical surface. In an effort to replicate the long-term depression (LTD) induced with pulsed protocols in invasive animal research and transcranial magnetic stimulation studies, we applied 100 ms pulses at 1.9 s intervals either in cortical-surface-anodal or cortical-surface-cathodal directions, with a placebo (sham) control. The results showed significant LTD of the motor evoked potential as a result of the cortical-surface-cathodal pulses in contrast to the placebo control, with a smaller but similar LTD effect for anodal pulses. The cathodal LTD after-effect was sustained over 90 min following current injection. These results support the feasibility of pulsed protocols with low total charge in non-invasive neuromodulation when the precision of targeting is improved with a dense electrode array and accurate head modeling.

## Introduction

Over the last two decades there has been a resurgence of interest in non-invasive transcranial electrical stimulation (TES) for the modulation of neural function in humans (Nitsche and Paulus, 2000). In addition to bringing the promise of electrical manipulation of the brain back to modern neuroscience, researchers have made important advances in understanding the underlying mechanisms at the macroscopic level (Stagg and Nitsche, 2011). Progress is also being made in understanding the effects of electrical currents at both mesoscopic and microscopic levels (Bikson et al., 2004; Kabakov et al., 2012; Ranieri et al., 2012; Rahman et al., 2013).

The current for TES can be direct current (DC or polarizing) or alternating (AC). Direct current can be applied in intervals as an “oscillatory” or variable manner, with similar effects as transcranial direct current stimulation (tDCS) in some studies (Groppa et al., 2010), and driving endogenous EEG rhythms, such as slow waves in sleep, in others (Marshall et al., 2011). More recently, it has been shown that transcranial pulsed current stimulation (tPCS) can also be used to alter cortical excitability (Jaberzadeh et al., 2014). The pulsed protocols are particularly important because they suggest the ability to draw from the literature on long-term depression (LTD) and long-term potentiation (LTP) with supra-threshold, pulsed protocols in animal studies (Froc et al., 2000; Bear, 2003) to improve lasting effects that may be more relevant for neurorehabilitation than the transient polarization of the cortex observed in many tDCS studies.

Exogenous current sources appear to affect neuronal excitability (and ultimately neural plasticity) in the same way as endogenous electrical fields generated by populations of active neurons (Frölich and McCormick, 2010), with both direct and alternating currents affecting neural activity by regulating up (firing) and down (quiescence) states. In addition to evidence that non-invasive neuromodulation alters immediate cognitive function (Wassermann and Grafman, 2005; Jacobson et al., 2012) some findings have suggested that LTD and LTP may be extended over several weeks (Reis et al., 2009). With the ability to induce long term changes in neural function, researchers have explored clinical applications, such as treatment of epilepsy (Fregni et al., 2006), stroke rehabilitation (Boggio et al., 2007), treatment of depression (Loo et al., 2012), and the specific topic of this special issue, pain management (Castillo-Saavedra et al., 2016).

Despite these advances, TES as a technology can still be regarded as being in its early stages, with many issues to still be resolved (Horvath et al., 2014). Because current flow cannot be focused, but rather follows the path of least resistance through the head tissues, an accurate model of electrode positions and head conductivity is required (Wagner et al., 2007). Furthermore, because current is likely to have different effects when aligned with the neuronal columns (normal to the cortical surface) than when crossing them (tangential flow; Bikson et al., 2004; Rahman et al., 2013), it is important to model the individual’s cortical geometry with cortical surface extraction from anatomical MRI (Li et al., 2016) in order to compute the components of induced current flow that are normal vs. those that are tangential. Moreover, there is now increasing interest in moving beyond the use of two large sponge electrodes, such as with the “high-definition” pattern of one source electrode surrounded by four sinks (Kuo et al., 2012), to improve precision of TES. Improving the specification of current density at the target, thereby computing the effective dosage, may be important to account for the considerable variability that is observed across individuals (Lopez-Alonso et al., 2014; Wiethoff et al., 2014). As described by Wiethoff et al. (2014) only about 36% of the participants showed the canonical pattern of anodal-facilitatory/cathodal-inhibitory after-effects that are typically assumed in the literature. Furthermore, the evidence of a non-linear relation between current dosage and measured after-effects for both motor (Batsikadze et al., 2013; Monte-Silva et al., 2013; Simis et al., 2013) and cognitive functions (Benwell et al., 2015) implies that consistency of treatment may be highly sensitive to dosage precision, even though underlying mechanisms that produce the non-linear effects may differ between motor and cognitive functions.

The goal of the present research was to evaluate the feasibility of more effective neuromodulation through improving targeting precision with a number of technical advances and use of a slow-frequency pulsed-stimulation protocol. We employ the standard protocol for assessing the effects of tDCS by targeting the hand area of the primary motor cortex and use of transcranial magnetic stimulation (TMS) as the cortical excitability probe. To minimize after-effect variability that may be attributable to previous technological and methodological limitations, in the present study for each participant we (1) identify the TMS motor hotspot through use of a neuronavigation system, (2) construct a high-resolution electric head model to determine direction of current distribution at the cortical surface, (3) select the optimal scalp electrode montage for current injection based on the reciprocity theorem, and (4) use dense-sensor arrays and multiple current sources to optimize current flow to the targeted cortical region. These technological and methodological procedures enable us to account for variations in individual anatomy and ensure that the target region always has the intended radial current direction.

The slow-frequency pulsed electrical stimulation protocol was modeled after *in vivo* animal work indicating that supra-threshold, low-frequency (0.5–3.0 Hz) stimulation induces LTD (Froc et al., 2000; Bear, 2003). Such findings motivated the development of low-frequency TMS protocols that were then shown to produce depression of motor cortex excitability (Chen et al., 1997). Following on those findings, slow (0.5 Hz) pulsed repetitive TMS was then shown to reduce cortical excitability and decrease the frequency of seizures for up to 6 months in epileptic patients (Sun et al., 2012).

Jaberzadeh et al. (2014, 2015) showed that *sub-threshold*, pulsed stimulation with a duty cycle that approaches tDCS determines the level of corticospinal excitability. Although we are not aware of direct evidence of pulsed, sub-threshold stimulation modulating plasticity in the same way that has been demonstrated with supra-threshold, low-frequency pulsed stimulation studies, the evidence that tDCS induced plasticity are Ca^2+^ dependent (Stagg and Nitsche, 2011), like supra-threshold findings, and results from human TMS work lead us to hypothesize that, even at *sub-threshold* stimulation, low-frequency stimulation is the important factor. Specifically, *we hypothesize that sub-threshold low-frequency (0.5 Hz) pulses will produce consistent inhibitory responses, regardless of the direction of current.* Moreover, based on the first hypothesis, we also examine a second hypothesis: *total charge required to affect cortical excitability will be minimal, compared to levels required in previous tDCS (including pulse and oscillatory) studies.*

## Materials and Methods

### Participants

Twelve participants took part in the study and completed all five sessions. Participants were recruited from Electrical Geodesics Inc. (EGI) and the University of Oregon. All participants were screened for MRI and TMS contraindications prior to acceptance into the study. Ten participants were male and the average age was 37 (*SD* = 10) and all were right handed. No participants were excluded from the study for any reason, including non-canonical after-effect responses.

### Study Design

Institutional Review Boards (IRB) at EGI and the University of Oregon approved the human subject use protocol for the present study. Prior to each session, participants provided informed consent. The study required five sessions (1 day per session) to complete. The first involved MRI acquisition and took approximately 20 min per participant. The second session involved TMS mapping to determine the location in primary motor cortex that elicited the strongest (i.e., “hotspot”) index finger EMG response. The second session also involved application of the HydroCel GSN (HC GSN) and Geodesic Photogrammetry to determine the 3-dimensional position of each sensor (see below). After the second session, the electric head model and stimulation plan were constructed. The three remaining sessions involved either a placebo (sham), anodal, or cathodal protocol; the order was counter-balanced across participants using a 3 × 3 latin square design. Participants were informed that one of three stimulation sessions would be a placebo. Both participants and TMS operator were blind to the electrical stimulation condition for any given session. A minimum of 48 h separated the three electrical stimulation sessions (Mean = 10 days, *SD* = 11).

### Structural MRI

Structural MRI data were obtained in all participants for use with Neuronavigated TMS and construction of high-resolution electrical head models. T1-weighted scans were obtained using Siemens’ MPRAGE sequence [repetition time (TR) = 2.5 s; echo time (TE) = 3.4 ms; flip angle (FA) = 8°] with a 1 mm × 1 mm × 1 mm resolution covering 256 voxels in each spatial direction. Data were acquired in Siemen’s 3T Skyra (Siemens Medical Systems, Erlangen, Germany) scanner using a 20-channel, head-neck coil. Sequence time was approximately 10 min. Foam padding was used to minimize head movements, and all participants were highly cooperative.

### Transcranial Magnetic Stimulation

Transcranial magnetic stimulation was accomplished with the Brainsight neuronavigation system (Rogue Research, Montreal, QC, Canada) and the STM9000 TMS system (EBNeuro, Florence, Italy). Each participant’s T1 MRI data was used in the Brainsight system to reconstruct the scalp surface. The scalp surface was registered with the participant’s head for each TMS session. A figure-of-eight coil (diameter of one winding = 70 mm, peak magnetic field = 3.2 T) was used and all stimulation employed a monophasic pulse. Motor evoked potentials (MEP) were recorded from the right first dorsal interosseous (FDI) muscle with Ag-Ag Cl electrodes arranged in the belly tendon montage and connected to Brainsight’s integrated EMG module. The MEP signal was bandpass filtered between 16 and 470 Hz, amplified by 4444, and digitized with a 12 bit ADC at a 3 kHZ sampling rate.

**FIGURE 1:**
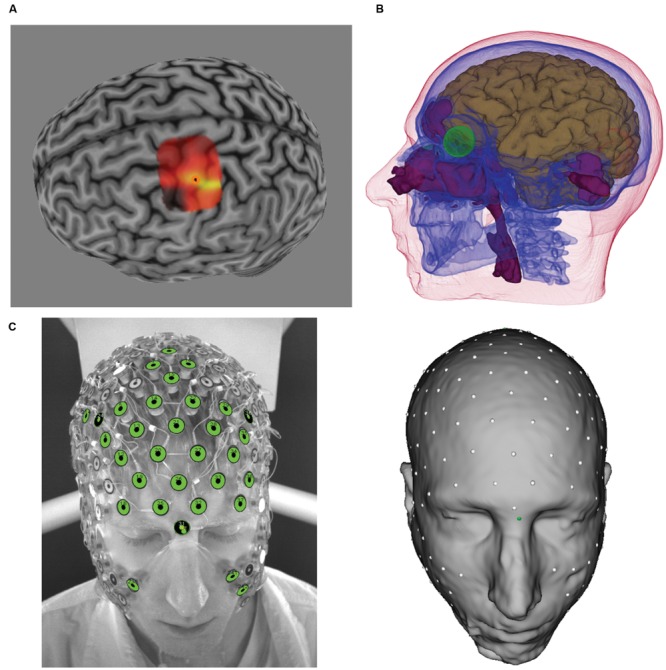
**(A)** Transcranial magnetic stimulation (TMS)-induced motor evoked potentials (MEP) heat map over M1. Brighter colors represent larger MEP amplitudes. Orange circle denotes “hotspot.” **(B)** Tissues identified from MRI and atlas CT, including brain (brown), skull (blue), eyes (green), air (purple), and scalp. Not shown are white matter and CSF. **(C)** photographic image from GPS (left) and sensors registered to the scalp surface (right).

To identify the location that elicited the strongest index finger response, the cortical surface was characterized with Brainsight’s curvilinear reconstruction method, and the hand region was identified using anatomical landmarks (Yousry et al., 1997). Once the hand region was identified, a virtual 5 × 5 grid (5 mm spacing between each position) was placed over the region to systematically guide TMS coil placement. For each location, the TMS coil was positioned with the handle pointing 45° posterolaterally relative to midline. At each site, two monophasic pulses (separated by at least 6 s) were delivered, with participants instructed to keep the hand and fingers in a relaxed state, and the MEP was qualified as a peak-to-peak measurement. After sampling of all of the grid positions, if the hotspot was at the edge of the grid, the grid was moved such that the hotspot was at the center of the grid and mapping was performed once again. Once this was completed, 3–5 additional pulses were applied over the hotspot for verification. A sample of the MEP amplitude map and identified hotspot is provided in **Figure 1A**.

Resting motor threshold (rMT) was defined as a percentage of TMS power output required to elicit MEP amplitudes of 50 μV in 5 out of 10 stimulation pulses, and this was determined with the hand and fingers in the relaxed state. Baseline MEP was specified as the TMS power output of 130% of rMT and the MEP amplitude (peak-to-peak) was specified as the average MEP amplitude of 10 stimulation pulses (each separated by at least 6 s). On average, this translated to 75% of maximum machine output. In certain participants, 130% of rMT still produced MEPs that were below 1 mV. In order to allow MEP decrement due to TES (i.e., minimize potential for floor effects), in these participants TMS power was increased to achieve an MEP average of 1 mV over 10 stimulation pulses. In some participants, 1 mV MEP could not be obtained even as we increased the power output up to 87%, and we accepted the MEP amplitude at 87% (arbitrarily set limit) as the baseline. On average, this was equal to 122% of rMT in these participants. Across the 12 participants, the average baseline MEP amplitude across all three sessions (see Procedure) was 0.97 mV (*SD* = 0.35).

### High-Resolution Electrical Head Models

Each voxel of the structural MRI data was segmented and classified into seven tissue types using the Modal Image Pipeline (EGI, Eugene, OR, USA): eyeball, flesh, skull, cerebral-spinal fluid (CSF), gray matter (GM), white matter (WM), and air. Because the skull is the most electrically resistive tissue, it is important to model, and yet bone can not be accurately obtained from MRI data. To estimate the skull, an atlas skull model derived from CT (1 mm × 1 mm × 1 mm) was non-linearly warped to the participant’s MRI tissues (using the other tissues as a guide). Detailed information about tissue segmentation and CT warping procedures is described in Li et al. (2016), and a complete characterization of the various tissues from these procedures are illustrated for one participant in **Figure 1B**.

To describe current flow from the cortex to the scalp, the cortical surface was first characterized through the use of triangular meshes, which were then parceled into patches of approximately equal size. All models used in the present study contained 1200 dipole patches per hemisphere, with each patch ∼1 cm^2^ in size. For each patch, perpendicular directions of vertices within the patch were averaged to derive the average, perpendicular orientation for that cortical patch. This average, perpendicular orientation is used to describe the direction of current flow. Electrode sensor positions of the 256-channel HC GSN 100 (EGI, Eugene, OR, USA) were digitized using the Geodesic Photogrammetry System (GPS, Russell et al., 2005, EGI, Eugene, OR, USA). The digitized sensor positions were then registered, using the Modal Image Pipeline, to the scalp surface of the head model, and the registration was verified against the photographic images (see **Figure 1C**).

From the complete head model, a lead-field matrix (LFM), which describes the propagation of current from each cortical patch to each sensor position, was computed using the finite difference method (FDM, Salman et al., 2015). The following conductivity values (in Siemens/meter) were assigned to each tissue type and are based on previously reported literature values: Eyeball = 1.5, Scalp = 0.44, Skull = 0.018, CSF = 1.79, GM = 0.25, WM = 0.35, and Air = 0.0 (Ferree et al., 2000). The total time required for construction of the high-resolution head models from MRI to completion of the LFM took approximately 60 min per participant.

### Selection of Optimal Current Injection Electrodes

Present approaches to targeting the primary motor cortex with TES employ the standard M1-contralateral supraorbital placement with two large electrode patches. This standard placement is a limitation because current paths and cortical distribution are estimated based on scalp placement, but the spatial relation between electrode on the scalp and underlying cortex does not accurately characterize current flow through the head. Therefore, this approach can not ensure that current will be optimally delivered to the intended target. Accurate head models are required for selection of optimal current injection electrodes for each individual (Wagner et al., 2007). In the present study, selection of current injection electrodes were performed using high-resolution head models and the neuronavigated TMS results.

In order to select the optimal current injection electrode montage and determine the appropriate amount of current to deliver through each of the active electrodes for a given target, we rely on the Lorentz reciprocity relating current densities at differing points and their electromagnetic fields in a complex resistive volume in the Rayleigh-Carson formulation, which assumes that all current sources have compact support (for detailed information see Tai, 1992). This theorem can be extended to analysis of linear passive electrical networks (King, 1963), and further applied to EEG by relating the electric field at the cortical dipole location created by injecting a current on the scalp with the electric potentials at the scalp injecting points caused by the same dipole (Rush and Driscoll, 1969; Malmivuo and Plonsey, 1995; Nunez and Srinivasan, 2006). However, only recently has it been realized (Tucker, 2003; Salman et al., 2015; Fernandez-Corazza et al., 2016) that the reciprocity principle can be used for efficient computational solution for EEG source analysis and TES optimization. Specifically for TES, the reciprocity principle dictates that injection of the given current amplitude based on the scalp voltage field produced by a dipole at the target location maximizes the directional current density on the target location. To implement the reciprocity principle in our Geodesic Transcranial Electrical Neuromodulation (GTEN) Planning Module (EGI, Eugene, OR, USA) together with safety constraints, we identify the scalp topography and then shape the injecting current patterns in accordance with the scalp voltage amplitudes around the positive and negative ends of the voltage field. To do so, we first assign the number of source (anode) electrodes, N, and sink (cathode) electrodes, M, to use for current delivery. We then sort the electrodes according to the voltage derived from the lead-field projection from dipoles representing a given cortical target to the scalp, assigning the electrodes with the N largest voltages to be sources and those with M largest in absolute value negative voltages to be sinks. These electrode values are then normalized such that the largest source voltage is assigned a weight 1.0 and the largest sink voltage is assigned a weight -1.0. We then calculate the current at each electrode by multiplying each electrode’s weight with the maximum allowable current per channel. These values are summed to ensure that the total anodal and cathodal currents sum to 0.0. If this is not the case, the current values are re-normalized using the smaller of the two values to ensure all safety criteria are strictly adhered to. A final normalization is then used to ensure that the total current delivered does not exceed the total current requested by the plan, or by safety constraints, whichever value is smaller.

**FIGURE 2:**
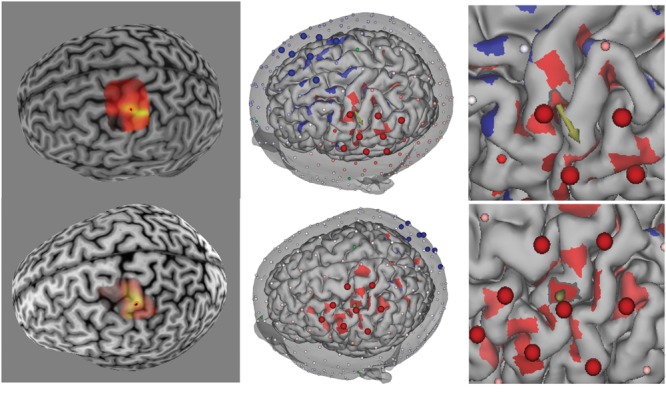
**(Left)** TMS-induced MEP heat map over M1 for two participants (top and bottom rows). Brighter colors represent larger MEP amplitudes. Orange circle denotes “hotspot.” **(Middle)** Reciprocity-based optimized selection of current injection electrodes for hotspot targets. Shown are eight anode (large red electrodes) and eight cathode (large blue electrodes). Note that the electrode montage is substantially different between the two participants because of the cortical geometry of the target (cortical patch with outline and arrow pointing in the average direction for the given cortical patch). Color intensity on the cortex represents current density normal to the cortical surface (red = anodal current direction, blue = cathodal current direction). Note that the current density was thresholded to remove the lowest 50% values in order to highlight the locations of high current density. **(Right)** Zoomed in view of the hotspot location to show the normal orientation (arrow) of the target (hotspot).

In the present feasibility study, we used a 16 channel prototype of the GTEN 100 system, such that the total number of electrodes used for each participant was eight anodes and eight cathodes. Maximum current at any given electrode (1 cm^2^) was limited to 200 μA. Given the weighting scheme described, this resulted in variable total current for each participant (mean = 1.16 mA, *SD* = 0.19) given the set number (eight) of electrodes. The average current density across all electrodes and participants is 0.15 mA/cm^2^ (*SD* = 0.02). Two examples from this procedure are illustrated in **Figure 2**.

### Transcranial Electrical Stimulation

Pulsed, direct current was applied using the prototype GTEN 100 system (EGI, Eugene, OR, USA) with the 256-channel HC GSN 100, which is an evenly spaced network of Ag-Ag Cl electrodes. The GTEN 100 has a double-fault Sentinel Circuit^®^ that monitors the sum total current such that the total current cannot exceed a 2 mA limit.

With targeting formulated mathematically through use of the reciprocity principle (as described above), the targeting is achieved in GTEN 100 via hardware that drives multiple constant current circuits to be balanced in the presence of multiple electrode impedances that are changing in time (through current-induced electroosmosis, iontophoresis, and electroporation of the electrode-skin interface). GTEN uses a proprietary balancing circuit, the AccuCharge Circuit^®^, capable of maintaining the designed balanced source-sink configurations over time.

Elefix conductive paste (Nihon Kohden, Tokyo, Japan) was mixed with over-the-counter lidocaine cream (5%) and used as the conductive material between the electrode and scalp. All electrode starting impedance were below 100 KΩ. Due to the iontophoretic mechanisms (Prausnitz, 1996), lidocaine was delivered to minimize physical perception of current stimulation at the scalp (Saliba et al., 2011), and electrode-scalp impedances were reduced over time as well (Oh and Guy, 1995). Note that with the constant current multichannel AccuCharge Circuit, the desired current level is maintained even as impedances drop with continued skin hydration. The stimulation protocol consisted of individual pulses (100 ms duration) at 0.5 Hz for 17 min.

Cathodal and anodal stimulation used the same electrode montage for each participant with current direction reversed. In addition to cathodal and anodal stimulation, a placebo condition was also employed in the present research. To minimize current flow to the targeted region while still maintaining potential for sensory perception associated with current flow in the placebo condition, stimulation used the same anodal-cathodal electrode clusters employed in the non-placebo conditions with the following modification. First, within each anodal and cathodal cluster, the electrode with the lowest current level used in the actual stimulation conditions were selected and the closest electrode neighbor was used to pass current. Therefore, in each cluster there was one pair (one anode and one cathode), and current (100 μA per pair and 200 μA total) passed through these pairs are prevented from penetrating deeply to affect the targeted region. Second, current was only delivered for five pulses (over 10 s) to further reduce the likelihood of any charge accumulation in the targeted brain region.

### Procedures

In sessions 2–5, participants were seated comfortably in a TMS chair (Rogue Research, Montreal, QC, Canada). Across sessions 2–5, participants were always scheduled for the study at the same time of day. Sessions 3–5 (the experimental electrical current stimulation sessions) started with the determination of MEP baseline followed by application of the Sensor Net and electrode-scalp impedance verification of the 100 KΩ threshold (Net Station 5.0, EGI, Eugene, OR, USA). Application of the Sensor Net in sessions 3–5 was guided by sensor positions in the 11 GPS images acquired in the second session (hotspot mapping). The GPS images provide 11 different views of the Sensor Net on the participant’s head. Following this procedure ensures that the sensors for a given session maintains the original sensor positions used to create the head model and stimulation plan.

Each electrical stimulation session (including placebo) started with a 30-s direct current conditioning period in one direction followed by another 30 s period in the opposite direction to facilitate the uptake of lidocaine through iontophoresis. The current level for each electrode was 50 μA (400 μA total). This brief duration of stimulation (with 1 mA) has been shown not to significantly affect neuronal excitability (Nitsche and Paulus, 2000). Next, current was delivered in 0.5 Hz pulses for 17 min, unless it was a placebo session. During this time, participants were instructed to sit comfortably in the TMS chair with their eyes open and hands and fingers in a relaxed state.

Participants were asked if they felt sensations (tingling, poking, burning, heating, and itching) during the direct current conditioning period, and during the pulse stimulation, participants were also asked if they felt any sensation at the following intervals: at start of stimulation and 4, 9, 13, and 17 min after start of the pulse stimulation. Upon cessation of stimulation, the HC GSN 100 was quickly removed (about 15–30 s) and TMS MEPs were immediately sampled followed by measurements at 5-min intervals for 30 min and at 60 and 90 min. Between the immediate to 30 post-stimulation measurement interval, participants remained seated in the TMS chair. After 30 min, participants were allowed to leave the room and return for the two remaining intervals. At each measurement interval, 12 stimulation pulses (separated by at least 6 s) were applied and the smallest and largest MEP amplitudes were excluded prior to averaging the remaining 10 MEPs.

## Results

### Report of Sensation during Current Stimulation

None of the participants reported adverse effects from participation in the study. Across the 72 (12 participants × 3 sessions × 2 polarities), 30-s current condition blocks (i.e., prior to pulse stimulation), participants reported sensations in 67 conditioning blocks. During pulse stimulation, participants reported feeling sensations in 21 blocks at the start of stimulation, 12 blocks after 4 min of stimulation, eight blocks after 9 min of stimulation, eight blocks after 13 min of stimulation, and three blocks after 17 min of stimulation. These data show that the conditioning period used for lidocaine delivery was effective in reducing sensations produced by the current by approximately 33%, and after 4 min of pulse stimulation, sensation was eliminated in approximately 66% of the sessions. By the end of the study, only one participant continued to report any sensation; this participant continued to experience slight sensations in all three sessions (including placebo). However, in the placebo session, pulses were only delivered for the first 10 s. No other participant reported sensations in the placebo session beyond the 1st minute after stimulation.

Only three participants reported experiencing phosphenes during pulse stimulation, and then only during anodal and cathodal sessions (and not for placebo). In these participants, the current injection electrode configuration included more frontal electrodes (e.g., top row in **Figure 2**), suggesting that they experienced retinal phosphenes. All of these three participants also correctly identified the placebo condition. An additional five participants were also able to identify the placebo condition; only four participants were not able to identify it.

### Modulation of Cortical Excitability

The first hypothesis was that TES applied at 0.5 Hz would produce a reduction in MEP amplitude, relative to baseline, regardless of the direction of the current. Of particular importance is that the polarity of the current is not defined by the direction of the current at the scalp (i.e., over primary motor cortex) but rather by the direction of current at the cortical surface of the target region as determined by each participant’s head model. Therefore, there is no ambiguity concerning cortical current direction, as would be the case without a model and the ability to optimize the stimulating electrode configuration.

**FIGURE 3:**
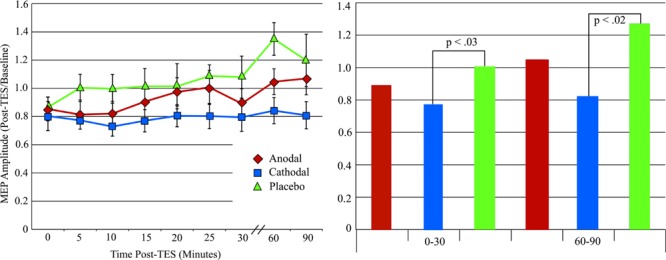
**(Left)** Motor evoked potentials amplitude changes after tPCS with error bars (standard error of the mean). **(Right)** MEP amplitude changes after tPCS grouped by time after stimulation.

**FIGURE 4:**
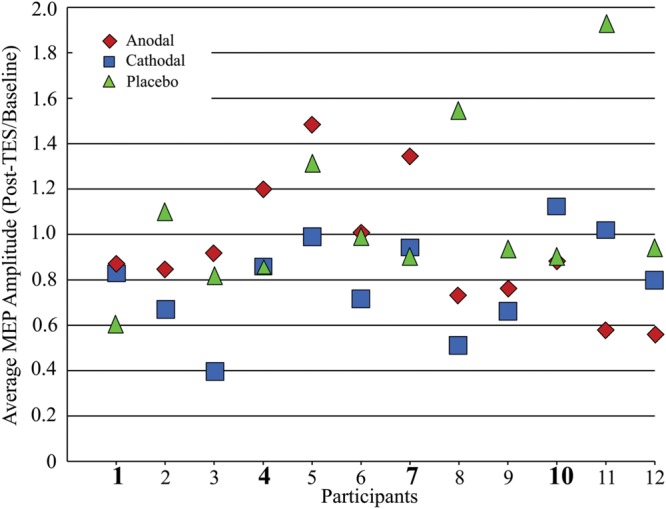
**Average MEP amplitude changes after tPCS plot for all 12 participants.** Participant numbers in bold highlight those participants with cathodal MEP amplitudes equal to or greater than placebo MEP amplitudes.

**Figure 3** shows the average MEP (as a percentage of the baseline MEP) for each condition. Consistent with our hypothe sis, over the post-stimulation course MEP amplitude for both cathodal and anodal stimulation protocols were reduced compared to placebo, with cathodal stimulation producing a larger reduction. Based on our hypothesis, we performed two one-tailed, paired *t*-test comparisons across the entire post-stimulation period: Anodal vs. Placebo and Cathodal vs. Placebo. The results showed that the difference between anodal stimulation and placebo was not significant, although the mean MEP amplitude after anodal stimulation also decreased (mean MEP amplitude relative to baseline = 0.93, *SD* = 0.08) relative to placebo (mean MEP amplitude relative to baseline = 1.1, *SD* = 0.36). In contrast, cathodal stimulation produce a significant reduction (mean MEP amplitude relative to baseline = 0.79, *SD* = 0.21) compared to placebo, *t*(11) = -2.41, *p* < 0.02. To explore the time course of the cathodal stimulation effect, we compared the post-stimulation MEP amplitude against MEP placebo amplitude for 0–30 and 60–90 min intervals. Paired *t*-test revealed that the difference was significant for the 0–30 min interval, *t*(11) = -2.26, *p* < 0.03, and 60–90 min interval, *t*(11) = -2.43, *p* < 0.02. As can be seen in **Figure 3**, the placebo condition showed a large increase at 60 and 90 min. Examination of the data showed that this increase was mainly due to one participant (11, see **Figure 4**). Therefore, we performed an analysis of the 60–90 min interval with this participant excluded to confirm the result. The paired *t*-test result showed that with this participant excluded, the effect is still significant, *t*(10) = -2.17, *p* < 0.03.

**Figure 4** shows the average MEP (across all post-stimulation measurement intervals) change relative to baseline for each participant. Of the 12 participants, eight demonstrated MEP amplitude decreases after cathodal stimulation in comparison to the placebo condition. The MEP response to anodal stimulation is more variable, with only five participants showing an amplitude reduction relative to placebo.

To test whether the cathodal stimulation produced more consistent inhibitory responses than conventional low spatial resolution methods, we compare our results to those reported by Wiethoff et al. (2014). Wiethoff et al. (2014) found that 22 out of 53 (∼41%) participants showed MEP amplitude reduction after cathodal stimulation (2 mA for 10 min). Because Wiethoff et al. (2014) did not include a placebo condition in their study, MEP changes were defined relative to only the pre-stimulation MEP baseline. As can be seen in **Figure 4**, relative to the baseline, in our sample there are a total of 10 participants who have inhibitory responses. Given the sample size, we computed an exact goodness-of-fit test. The test revealed that the observed distribution (i.e., more participants responded with a reduction in MEP amplitude) is indeed significantly different, *p* < 0.01 (one tailed).

We also performed an exact goodness-of-fit test to determine whether the number of participants showing an inhibitory response to anodal stimulation differed from the 26% reported by Wiethoff et al. (2014). In our sample, eight participants showed reduced MEP amplitudes after anodal stimulation, and this is significantly different than expected from the findings of Wiethoff et al. (2014) *p* < 0.004 (one tailed).

### Total charge and Excitability Modulation

A second hypothesis was that total charge is not the critical factor for determining the effect of TES. Given the present study’s stimulation protocol, the total stimulation time (100 ms at 0.5 Hz over 17 min) is 51 s and the total charge is 59.16 milli-coulombs (see **Table 1**). A previous study by Nitsche and Paulus (2000) concluded that anodal tDCS must be applied for at least 3 min (at 1 mA) for significant MEP changes after current cessation. For cathodal stimulation, Nitsche et al. (2003) showed that 5 min of stimulation at 1 mA produced very short lasting (1 min) after-effects; by 5 min the MEP returned to baseline. Examination of the total charge reveal that the total charge is similar between anodal stimulation in the present study and that used by Nitsche and Paulus (2000). However, for cathodal stimulation, the total charge in the present study is approximately five times less than the level used by Nitsche et al. (2003), and yet it was still effective in reducing the MEP amplitude for at least 90 min after stimulation.

**Table 1:** Comparisons of transcranial pulsed current stimulation (tPCS) and transcranial direct current stimulation (tDCS) after-effects as a function of total charge.

	Modulation type	Polarity	Total current (mA)	Area (cm^2^)	Total duration (seconds)	Pulse duration (seconds)	Hz	Total current injection time (seconds)	Total charge (mCoulomb)	Findings
Present study	tPCS	Anodal/Cathodal	1.16	8	1020	0.1	0.5	51	59.16	Significant MEP decrease for cathodal stimulation
Nitsche and Paulus, 2000	tDCS	Anodal	1.0	35	60	NA	NA	60	60	No significant change
Nitsche et al., 2003	tDCS	Cathodal	1.0	35	300	NA	NA	300	300	Significant MEP changes lasting for only 1 min after stimulation (returned to baseline at 5 min)

*Stimulation parameters are also listed for comparison.*

**Table 2** also compares total charge used in the present study with two studies that use similar pulse-like stimulation protocols. Groppa et al. (2010) applied slow oscillatory tDCS (so-tDCS) using both anodal and cathodal currents at two different current levels and assessed its affect on cortical excitability. They found that post-stimulation MEP amplitudes were only affected by tDCS and the higher current level so-tDCS protocol. Jaberzadeh et al. (2014) only used anodal stimulation but employed both tDCS and tPCS. For tPCS, these authors manipulated the inter-pulse interval while keeping the pulse duration and total charge constant. They found that short inter-pulse intervals produced significantly larger MEP responses compared to both the placebo condition and standard tDCS. However, long inter-pulse interval tPCS produced only a small and non-signifiant increase in MEP amplitudes.

**Table 2:** Comparisons of tPCS and so-tDCS after-effects as a function of total charge.

	Modulation type	Polarity	Total current (mA)	Area (cm^2^)	Total duration (seconds)	Pulse duration (seconds)	Hz	Total current injection time (seconds)	Total charge (mCoulomb)	Findings
Present study	tPCS	Anodal/ Cathodal	1.16	8	1020	0.1	0.5	51	59.16	Significant decrease for cathodal
Groppa et al., 2010	tDCS	Anodal/ Cathodal	0.75	12	600	NA	NA	600	450	Significant increase/decrease
	so-tDCS		0.75	12	600	tACS	0.8	tACS	225	No significant change
	so-tDCS		1.5	12	600		0.8		450	Significant increase/decrease
Jaberzadeh et al., 2014	tDCS	Anodal	0.7	24	600	NA	NA	600	420	Significant increase
	tPCS (short)		1.5	24	300	0.5	1.8	272.7	409.1	Significant increase
	tPCS (long)		1.5	24	600	0.5	0.9	260.9	391.3	No significant change

*Stimulation parameters are also listed for comparison.*

In summary, with improved targeting and the brief slow pulse protocol, the present methods achieved significant LTD with much lower total charge than required in previous studies with conventional low spatial resolution tDCS, so-tDCS, and tPCS methods.

## Discussion

Participants in the present feasibility study did not report any adverse side effect from the tPCS protocol using the dense-array electrode configuration. Given that the current density for the electrode with the maximum current (200 μA/cm^2^) is greater than those used in previous studies, it is important that participants did not report uncomfortable sensations during the conditioning period (before lidocaine became effective at reducing sensations). The time-course of reported sensations showed that lidocaine became increasingly effective at reducing reports of sensations. It is expected that with constant current (rather than tPCS), the time course would be accelerated because of a more sustained iontophoretic application of the lidocaine.

Our use of lidocaine was intended to minimize discomfort over the full session; even if participants report the stimulation is not painful in the beginning, it may become uncomfortable when continued for many minutes. Waiting longer (∼ 20 min) after lidocaine application would minimize sensation. In the present experiment, 8 out of 12 participants correctly guessed the placebo condition, but this was due in part to the greater experience of phosphenes during tPCS compared to the placebo condition.

### Modulation of Cortical Excitability

An important theoretical question for the present approach is whether, given improved spatial targeting of the oriented cortex, it is possible to manipulate cortical plasticity through more complex temporal parameters of activity-dependent neural plasticity. The Bienenstock–Cooper–Munro theory describes how LTP and LTD occur as a function of a modification threshold (for a historical overview see Bear, 2003). According to this theory, LTP and LTD occur when presynaptic activity is associated with post-synaptic activity that is above or below a certain threshold, respectively. The physiological mechanism of the modification threshold has been shown to be the level of Ca^2+^ flux, which is controlled via voltage gated channels, into the postsynaptic cell (Mulkey and Malenka, 1992). In slice preparations, the level of Ca^2+^ influx can be electrically manipulated through variations in the rate of stimulation (Mulkey and Malenka, 1992; Kirkwood et al., 1993), with low-frequency stimulation producing LTD. These findings motivated human studies that show low-frequency stimulation using TMS produce a reduction in motor cortex excitability (Chen et al., 1997). Based on this evidence, we predicted that low-frequency tPCS also would result in reduced cortical excitability, regardless of the current direction. Moreover, we also predicted that the after affects of low-frequency tPCS should be more consistent across subjects.

Consistent with these predictions, we found that both anodal and cathodal pulsed stimulation at 0.5 Hz produced inhibitory after-effects that were below baseline and, more importantly, below the level observed for the placebo condition. Moreover, when we examined the proportion of participants who exhibited inhibitory responses to both anodal and cathodal stimulation against previously reported proportions (Wiethoff et al., 2014), the difference was significantly greater in the present study.

In the present study, we found that the reduction in MEP amplitude was significantly different from placebo only for cathodal stimulation, raising the theoretical question of why the directional polarization (surface-cathodal) of the cortex is relevant for the induction of LTD by slow pulses. In this regard, the anodal and cathodal pulses were not equivalent, suggesting polarization with respect to the cortex is important. The observation of relatively weak anodal after-effects has been reported previously in several studies with tDCS. In a study by Dieckhöfer et al. (2006) examining the effects of tDCS on the N20 SEP component from primary somatosensory cortex, only cathodal stimulation produced a significant N20 amplitude reduction. Anodal after-effects were not observed. A recent tDCS study with rabbits showed a similar effect: cathodal stimulation of somatosensory cortex reduced N1 amplitude, whereas anodal stimulation did not (Márquez-Ruiz et al., 2012). Rogalewski et al. (2004) found that only cathodal stimulation over sensorimotor cortex reduced tactile discrimination performance (up to 7 min post stimulation) when compared to placebo, whereas anodal stimulation had no effect. Antal et al. (2004) noted that cathodal tDCS after-effects lowered beta and gamma power after stimulation in response to a visual stimulus whereas anodal tDCS had no effect.

Sommer et al. (2013), using TMS, induced orthodromic (analogous to anodal TES) or antidromic (analogous to cathodal TES) current flow with 1 and 5 Hz TMS pulses. These researchers reported that for 1-Hz, monophasic pulse protocol, antidromic current flow produced significant inhibitory after-effects and orthodromic current flow did not (note that 5-Hz monophasic stimulation did not produce any significant after-effects). These findings are consistent with our findings of a significant after effect only for cathodal stimulation (see below). It is worth noting that Sommer et al. (2013) also found significant facilitatory after-effects for 1-Hz stimulation when a biphasic pulse was used. How this latter finding may be related to the present results (or the model discussed below) is unknown.

Transcranial current delivery produces diffuse current flow and thus affects synapses and cells distributed across all cortical layers (Fritsch et al., 2010) and over a broad area of cortex (including beyond primary motor cortex, Stagg and Nitsche, 2011). Afferent inputs not perpendicular to the cortical surface are also affected (i.e., they can also be polarized or depolarized) because of the substantial tangential current component of the current flow (Rahman et al., 2013). Therefore, the observed after-effects represent the summed influence on neuronal compartments over a relatively large area. When current flow is estimated for a specific cortical target, as with the high resolution electric head model, there is an overall polarization of the cortex, where the large vertical pyramidal neurons experience somatic depolarization and hyperpolarization in the presence of cortical-surface-anodal and cortical-surface-cathodal current fields, respectively. Therefore, the asymmetry of anodal and cathodal stimulation in the present study suggests a differential effect of the direction of polarization on the neuronal populations of the motor cortex.

Recognizing that LTD and LTP effects are mediated by Ca^2+^ influx, it is important to consider a regulatory mechanism [Ca^2+^-activated small conductance (SK) channels] at the dendritic spine that prevents over activation produced by Ca^2+^ influx. Tigaret et al. (2016) showed that LTP at the dendritic spine depends on overriding this regulatory mechanism by slow-acting group 1 metabotropic glutamatergic receptors (mGluR1). Because the apical dendrites are hyperpolarized and depolarized in anodal and cathodal electrical fields, respectively, and because SK channels (Maciaszek et al., 2012) and mGluR1 receptors (Luján et al., 1996) are densely distributed at the dendritic spines (and sparsely at the dendritic trunk and soma), the mechanisms of plasticity induction may be biased toward the dendritic spine compartment of the neural network. As noted by Gee and Oertner (2016), Ca^2+^ flux in dendritic compartments and the interaction with Ca^2+^ levels in the soma must be considered for a complete picture of how long term plasticity is induced. Although this interaction is not fully understood at present, it may be that the more powerful effect of cortical-surface-cathodal current on the dendritic region may explain why this direction of polarization is more effective in producing both tDCS and tPCS after-effects.

Thus, in a static DC surface-anodal electric field, there is very little change in activity (i.e., minimal Ca^2+^ flux) in the apical compartments. The summed effect reflects plasticity changes centered on the somatic compartment (where activity is summed in the initial segment). For surface-cathodal current, changes occur primarily in the apical compartment, and the soma’s integration of activity is affected by the polarized state induced by a cathodal electric field. Because the effects are primarily in the apical dendritic compartment, surface-cathodal current may be more effective due to the dense distribution of plasticity inducing mechanisms (such as mGluR1 and SK channels) in this compartment. Consistent with this reasoning is the observation that only cathodal tDCS significantly modulates TMS input-output curves (Nitsche et al., 2005; Stagg and Nitsche, 2011). The input-output curve metric is believed to reflect the activation of corticospinal tract neurons as well as intracortical neurons over a wide area, which would engage feedforward and feedback connections between neurons at superficial layers and across the apical neuronal compartment. Also consistent with this reasoning are findings by Sommer et al. (2013), who showed the directionality of current flow induced by TMS is important to the induction of neuroplasticity. As noted above, these researchers demonstrated that when current flows from layer I (apical) to layer VI (basal), analogous to anodal electrical stimulation, plasticity changes were not observed. On the other hand, when current flow was directed from layer VI toward layer I (analogous to cathodal stimulation) plasticity changes were significantly demonstrated. Sommer et al. (2013) attributed the plasticity changes induced by antidromically flowing current to changes in excitability in the apical dendritic compartment.

Another possible explanation for the observed after-effect asymmetry for anodal and cathodal stimulation relies on the concept of homeostatic plasticity. Homeostatic plasticity describes the fact that neurons have mechanisms that restore baseline levels of neuronal function (Davis, 2013; Tigaret et al., 2016). Using this concept, it is plausible that low-frequency anodal pulsed stimulation indeed inherently produce facilitation but “over excites” the affected neurons such that homeostatic compensatory mechanisms are engaged and thus, result in low-level inhibitory responses. However, this appears unlikely. Given that (1) cathodal stimulation provided the same amount of current, (2) SK and mGluR1receptors that form part of the homeostatic compensatory mechanism are densest in the apical compartment, and (3) cathodal stimulation depolarizes the apical compartment, one would expect homeostatic mechanisms to be most sensitive to cathodal stimulation, and yet no compensation appeared to have occurred.

The variability of responses observed in previous research may be due to the poor precision of cortical targeting. Given the variability in participants’ anatomy and the use of large M1 electrodes vs. contralateral supraorbital stimulation electrodes, it is possible that current at the target area of the cortical surface (such as the finger area) is not of the desired polarity. Additionally, given the demonstration of non-linear effects of total charge on after-effects direction (Batsikadze et al., 2013; Simis et al., 2013; Benwell et al., 2015), the inability to account for total charge variations may be a significant factor contributing to the variable responses.

In the present study, we observed greater consistency of LTD across participants. By employing the reciprocity principle between EEG and TES with high resolution subject-specific head models, the optimal stimulating electrode montage was always individually adjusted such that current delivered to the motor cortex hotspot was maximized radially. Because of the very nature of the low-frequency pulsed protocol, total charge is relatively low. Moreover, low-frequency stimulation is a well-established protocol for inducing LTD because it directly affects the rate of Ca^2+^ influx, which in turn affects the cascade of neurophysiological events that induce LTD. In the present feasibility study, we did not manipulate these factors separately, so we cannot determine the extent to which these three variables contributed to the reduced variability.

### The Influence of Pulsed Stimulation vs. Total Charge on Cortical Excitability

We also predicted that total charge is not the critical factor in determination of the effectiveness of tPCS, and that brief pulses would be adequate. As shown in **Tables 1** and **2**, results from prior tDCS and so-tDCS studies suggest that total charge is important to the after-effects. In tDCS, low total charge produces little to no significant after-effects. In the so-tDCS study, Groppa et al. (2010) showed that anodal stimulation at 0.75 μA produced no significant after effects. However, keeping the duration the same but increasing the current to 1.5 mA produced significant after-effects for both cathodal and anodal stimulation.

Jaberzadeh et al. (2014) performed a study most similar to the present study. In their study, using anodal pulses that are 500 ms wide separated by short (50 ms, 1.8 Hz) or long (650 ms, 0.9 Hz) inter-pulse interval (changing total stimulation duration to keep total charge approximately equal), the authors found that cortical excitability was changed only for the short inter-pulse interval (longer duty cycle) protocol. The authors concluded that it is the inter-pulse interval that is important to the observed effects and not the total charge or pulse width, because these two variables were controlled to be approximately equal across the short and long inter-pulse interval protocols. Their conclusion that total charge is not the relevant factor for determination of significant after-effects is consistent with our proposal.

The explanation proposed by Jaberzadeh et al. (2014) for the lack of effect with the long inter-pulse interval protocol (43.5% duty cycle) was that this protocol prevented accumulation of charge. However, the inter-pulse interval employed in the present study is more than twice as long (1900 ms, 5% duty cycle), and yet participants showed significant after effects. Moreover, given that the frequency used by Jaberzadeh et al. (2014) is within the slow frequency range that should produce inhibition (i.e., LTD, Bear, 2003), it is surprising that these researchers observed facilitation for anodal stimulation, which is contrary to our findings. This discrepancy may be attributable to the pulse width difference between their study (500 ms) and our (100 ms) study. However, in a more recent study, Jaberzadeh et al. (2015) used a similar pulse width (125 ms), but with very short inter-pulse interval (50 ms, 5.7 Hz), and the results showed no significant after-effects. Given these results, it is likely that short pulse duration stimulation has to be coupled with low-frequency stimulation to induce significant after-effects, consistent with evidence from animal LTD studies (Froc et al., 2000) and TMS findings with low-frequency stimulation (Chen et al., 1997).

### Study Limitations and Future Directions

In the present research, we demonstrated feasibility of a pulsed protocol with low current when high resolution modeling of cortical targeting is employed, yet we did not include experimental manipulation of each of the potentially contributing factors. Future studies will be required to clarify the contribution of each variable. Although we showed that very low total charge can still induce significant after-effects, it is still possible that there exists a relation between total charge and magnitude as well as duration of after-effects using a tPCS protocol. Future studies should address this possibility. We, as well as Jaberzadeh et al. (2014), showed that the inter-pulse interval parameter appears to be important, and yet opposite effects are observed for anodal stimulation in the two studies; pulse width and frequency is the obvious variable that may contribute to the observed difference and this should be tested in future studies.

By considering the greater concentration of plasticity mechanisms at dendritic spines rather than at soma levels, we have suggested a model to understand the effects of the direction of polarization on cortical excitability that generates testable predictions. For example, one prediction is that faster frequency stimulation, known to induce LTP, will also show greater facilitation for cathodal stimulation pulses.

## Conclusion

In this feasibility study, we were able to implement a number of improvements in the spatial targeting of cortical-surface-normal electrical current to the specific finger motor area of each participant, and to observe that the slow pulse electrical stimulation was effective for the induction of LTD. As hypothesized, LTD was achieved with much lower total charge levels than are required for tDCS protocols. Although not significantly different from placebo in this sample of 12 participants, 0.5 Hz anodal tPCS also reduced cortical excitability. The consistency of reduced cortical excitability was greater across participants than has been reported in previous research. Finally, we proposed a model for how to understand the apparent asymmetry of anodal and cathodal stimulation effects of tDCS and tPCS. For non-invasive TES to contribute maximally to clinical applications particularly, it is clearly important to understand how to achieve reliable induction of cortical plasticity in each person.

## Author Contributions

PL, EMEA, and DT contributed to the design of the study. DT, EA, and ST contributed to the design of the Reciprocity algorithm. DR designed of hardware system for current injection. EMEA, AG, and PL were responsible for data acquisition and analysis. All authors contributed to the interpretation of the results and preparation of the manuscript. All authors acknowledge that they are accountable for all aspects of the work.

## Conflict of Interest Statement

Authors of this paper are employees of a commercial EEG company, Electrical Geodesics, Inc., and EGI holds several patents for technologies used in the present research including: US Pat. No. 7,840,250, No. 6,594,521, No. 7,190,826, and 8,478,011.
